# Clinical experience of using a novel extracorporeal cytokine adsorption column for treatment of septic shock with multiorgan failure

**DOI:** 10.1186/cc14210

**Published:** 2015-03-16

**Authors:** P Sathe, P Sakhavalkar, S Kumar, S Choudhary

**Affiliations:** 1 Ruby Hall Clinic, Pune, India

## Introduction

Severe sepsis and multiorgan failure (MOF) are major causes of death in the ICU. The extracorporeal cytokine adsorption column (ECAC; Cytosorb®, CytoSorbents Corporation, USA), a critical care focused therapeutic device, results in rapid *in vitro *and *in vivo *elimination of several key cytokines and prevents organ failure. Use of ECAC in patients with sepsis is a new area of research with insufficient data to promote large prospective RCTs. Studies published to date have shown promising results. We report our clinical experience with ECAC for severe sepsis/septic shock/MOF patients.

## Methods

A retrospective evaluation of ECAC in patients admitted to a tertiary ICU from November 13 to October 14 to analyze: clinical safety; selection of a subgroup of patients where it could be used; selection of timing for initiation; number of device filters required per patient; and selective markers to identify above initiation. Patients were managed with standard of care (SOC; antibiotics, vasopressors, i.v. fluids, sepsis dosed steroids) and ECAC as adjuvant therapy. Vitals, APACHE II and SOFA scores were measured.

## Results

Nineteen ICU patients (14 men, five women; 24 to 72 years; average ICU stay 10 days; average ventilator days 9) with APACHE II >17 (except one with dengue shock syndrome), SOFA score ≥11 (*n *= 16) and the majority having infection largely in the lung (*n *= 8; alone or with UTI and blood infection) followed by the abdomen (*n *= 4), UTI (*n *= 3) and others (*n *= 4) were given ECAC (total ECAC = 31). Predicted mortality (PM) was >40% in 16, >30% in two and <30% in two (tropical infections) patients. Duration of therapy was 6 hours (no. of ECAC = 18) and 8 hours (*n *= 4; no. of ECAC = 5) for the majority of patients. Overall, four patients (two with tropical infections and two with PM >40%) survived; three of them had were ECAC early (<24 hours of admission). The majority of patients (*n *= 11) who died could be given ECAC only once. Of patients who died, seven were given ECAC late (>24 hours). APACHE scores before and after ECAC therapy were available for eight patients who died; APACHE score decreased >5 points in five patients after single application of ECAC.

## Conclusion

ECAC can be used as adjuvant therapy in treatment of severe sepsis/septic shock/MOF. Our patients had high PM and four could be saved with use of ECAC. We could expect a better outcome if ECAC was used early (<24 hours) during treatment. However, future well-designed studies are needed to clarify the role of ECAC in patients with MOF/septic shock.

